# Latero-posterior directed migration of trunk to the concave side of the curve: a biomechanical principle in treating the three dimensional deformity of idiopathic scoliosis with a custom molded high profile TLSO

**DOI:** 10.1186/1748-7161-8-S1-P13

**Published:** 2013-06-03

**Authors:** SH Jang, JA Hutson

**Affiliations:** 1 Assistive Technology Department, Gillette Children's Specialty Healthcare, St. Paul, MN, USA

## Background

The biomechanical principles that guide orthotic treatment for Idiopathic Scoliosis (IS) are not fully defined, except for the ‘three point correction’ principle.

## Aim

This study aims to discover the underlying biomechanical principles, used by experienced orthotists, in treating the three dimensional deformity of IS, with a custom molded TLSO.

## Methods

Semi-structured individual interview, and focus group methodology , were the primary methods of data collection. Detailed descriptions of orthotic treatment, for a specified case example, were obtained from seven experienced spinal orthotists; participants held an average of 16.7 years experience in IS treatment. Sessions were audiotaped, transcribed and data was analyzed using a systematic approach to identify themes. Triangulation of data was completed.

## Results

Achieving a “balanced and aligned spine and trunk in all 3 planes” emerged as the primary biomechanical goal for all 7 participants (100%). The orthotists identified specific techniques of the treatment process such as:

• drawing an iliac-clavicle box on the PA x-ray • determining the location and the degree of forces and finding flexibility of the curve with a hand technique • reducing lumbar lordosis during casting• de-rotating the trunk • centering the upper torso at the axillas over the pelvis • creating space on the lateral posterior area of the concave side of curve and the mid-posterior area of the spine •carving the model to achieve desired forces • applying abdominal pressure• building a sternal extension and a trochanteric extension.

## Conclusion

To achieve the biomechanical goal, and re-align the 3-dimentional deformity of IS, orthotists apply de-rotational, anterior, and lateral translational forces on the lateral side of the convex curve, and create space on the side opposite the applied force. These factors result in a biomechanical principle called latero-posterior directed migration of the trunk to the concave side of the curve.
